# A systematic review on the effect of the COVID-19 pandemic on childhood immunisation programmes of West African countries

**DOI:** 10.4314/gmj.v58i2.8

**Published:** 2024-06

**Authors:** Osamudiamen C Obasuyi, Veronica A Obasuyi

**Affiliations:** 1 Ophthalmology Department, Irrua Specialist Teaching Hospital, Irrua Edo State; 2 Department of Internal Medicine, Irrua Specialist Teaching Hospital, Irrua Edo State

**Keywords:** COVID-19, immunization, vaccination

## Abstract

**Objectives:**

To investigate the effects of the COVID-19 pandemic on childhood immunisation programmes in West African Countries.

**Design:**

The study was a systematic review of available evidence of the impact of the COVID-19 pandemic on childhood immunisation programmes in West Africa

**Setting:**

An online literature search was conducted using PubMed, Embase, Scopus and Web of Science for all peer-reviewed longitudinal, descriptive, observational, prospective and retrospective studies on childhood immunisation programmes in West Africa published between January 2020 and May 2022

**Participants:**

All West African childhood immunisation programmes.

**Interventions:**

None

**Main Outcome Measures:**

Change in immunisation volumes during the COVID-19 pandemic

**Results:**

353 studies were identified during the literature search, and eight were included in this review. The studies comprised six quantitative studies, one mixed-method (quantitative/qualitative) study and one qualitative study. Changes to immunisation services ranged between 53% and 52% for MCV and Penta3 vaccines in Guinea, lasting longer than August 2020, to 0.3% and 1% in Liberia for BCG and MCV vaccines lasting no longer than May 2020. Factors contributing to the observed disruptions in vaccine coverage during the pandemic included the fear of contracting the virus expressed by caregivers and healthcare workers and general misinformation about the COVID-19 virus.

**Conclusion:**

While the changes were greater than 50% and lasted longer in some countries, they were brief and short-lived in others, emphasising that the COVID-19 pandemic's effect in each country differed.

**Funding:**

This work did not receive any external funding and was entirely self-funded

## Introduction

The expanded programme on immunisation (EPI) was birthed on the heels of the success of the smallpox vaccination programme.^1^ and integrated into routine maternal and childhood services, offering mobile and facility-based immunisation services. The EPI provides the Bacille Calmette-Guerin vaccine (BCG), the Oral polio vaccine (OPV), the Diphtheria-Pertussis-Tetanus Vaccine (DPT), the Measles Vaccine (MCV), and the tetanus vaccine.^2^–^5^ Furthermore, the EPI allowed the domestication of the vaccines provided in the scheme based on local and prevailing epidemiologic factors, which has led to the introduction of the Hepatitis, Yellow fever, Meningococcal and Pneumococcal vaccines.^6^,^7^ The EPI also allowed for the conduct of National immunisation days (NIDs), Supplemental immunisation activities (SIAs) and catch-up immunisation campaigns to supplement routine immunisation services.^8^,^9^

Monitoring and evaluation of immunisation programmes use selected indicators, such as Vaccination coverage, disease incidence and surveillance, vaccine quality control, cold chain and vaccine management, and immunisation safety, retrieved from the UNICEF Multiple Indicators Cluster Survey, Demographic health surveys, and the EPI cluster surveys.^10^–^12^ DPT vaccination is routinely used to estimate vaccine utilisation, coverage and access.^13^,^14^ DPT1 vaccine coverage indicates entry points into the EPI programme, while DPT3 measures the programme's ability to retain the children. DPT dropout rates of > 10% denote poor utilisation of vaccine services.^15^

Vaccines have become one of the most cost-effective public health interventions.^16^ Over 116 million children are vaccinated annually (86% of all children born), and the use of vaccines has expanded to cover over 20 diseases and is being used to control epidemics or pandemics.^17^

African regional DPT3 coverage was 52% in 2010, which increased to 76% by 2015, with 34% of African countries achieving greater than 90% national coverage^18^, and by 2019, global DPT3 coverage averaged 81.6 %^19^. Similarly, African regional MCV1 coverage increased from 53% in 2000 to 74% in 2015, with Twelve African countries (26%) achieving more than 90% national coverage. BCG vaccines currently have the highest coverage, and the Oral Polio vaccine has the lowest coverage, according to a study among 25 sub-Saharan countries^20^.

However, vaccine access globally is still inequitable, adversely affecting marginalised and poor populations. Over 13 million children are currently classed as “zerodose children” because they have not had a single dose of a potentially life-saving vaccine^16^, 75% of whom live in 14 Low to Middle-income countries(LMICs).^19^ Another 30 million children in these areas also do not receive the full complement of vaccination even when available^16^. Due to the poor distribution or access to vaccines, diseases that may have been on a downward trend have been resurgent. For example, vaccine-derived poliovirus is becoming a problem in many African countries.^21^, and the incidence of measles has doubled in the last three years (2019-2021).^21^

Initial reports of the effects of the COVID-19 pandemic indicated disruption to immunisation programmes in over half of the countries participating in a global WHO pulse survey.^22^–^24^ Furthermore, 13.5 million children missed doses of their vaccines in thirteen of the world's poorest countries^25^,^26^ amidst reports of fresh outbreaks of the wild poliovirus in Afghanistan and Pakistan.^27^ Vaccine shipment was reduced by 80%, and lockdown regulations disrupted the production of vaccines.^28^,^29^ Most countries repurposed pre-existing health systems to provide health care for COVID-19 patients, providing COVID-19 vaccinations to the most vulnerable.^22^–^24^ At the same time, some non-emergency healthcare services, like immunisations that relied on physical contact and movement, were deprioritised.^22^–^24^ For example, the WHO initially recommended suspending immunisations in the first few months of the COVID-19 pandemic because it feared the virus would spread during routine immunisations.^30^ Furthermore, the misinformation about COVID-19 and its treatment may have affected many caregivers' and mothers' desire to use vaccination services.^22^–^24^ Combined with vaccine hesitancy in some communities, the desire for immunisation services may have dropped further.^31^–^34^

Given the fragility of health systems in West Africa and the struggle to achieve good childhood immunisation coverage, the risk of severe disruption to childhood immunisation programmes due to the COVID-19 pandemic exists. This review aims to quantify the COVID-19 pandemic's effects on West African immunisation programmes and conceptually synthesise why the pandemic had such an effect.

This review aims to determine how the COVID-19 Pandemic in 2020/21 affected childhood immunisation programmes in West Africa.

Specific objectives include:

1. To analyse the volume and trends of immunisation in the West African region pre-COVID-19 and compare these results with those obtained during the COVID-19 pandemic in 2020.
2. To explore why the COVID-19 pandemic may or may not have affected immunisation programmes in West African countries.

## Methods

The study was a systematic review without meta-analysis(SwiM) of all peer-reviewed longitudinal, descriptive, observational, prospective and retrospective studies on childhood immunisation programmes in West Africa published between January 2020 and May 2022. The COVID-19 pandemic had its most disruptive impact on healthcare in 2020, and this period was selected to enable the review to capture as many reports/studies on the subject as possible, taking into account the varying length of time for peer review and publishing.

**Figure 1:**
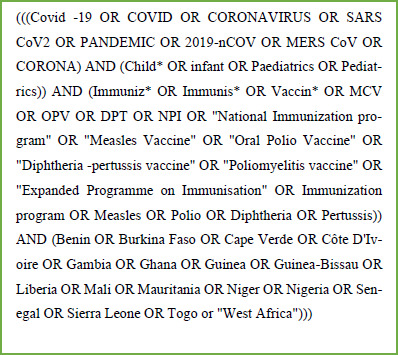
Search Strategy showing key search words and combinations

The protocol for this study was based on the Preferred Reporting Items for Systematic Reviews and Metaanalysis Protocols-PRISMA-P.^35^ A preliminary search was conducted using the PubMed database to validate the premise and availability of peer-reviewed articles covering the subject. Subsequently, a defined search strategy was designed (Figure 1).

### Inclusion criteria

All peer-reviewed longitudinal, descriptive, observational, prospective, and retrospective studies published between 2020 and May 2022 that studied or described the effects of the COVID-19 pandemic on childhood immunization programmes in West Africa were deemed eligible.

Also included were studies that described or studied the effects of the COVID-19 pandemic on vaccination coverage and the utilisation of these services directly via vaccination numbers and trends by comparing pre and post-COVID-19 values and allowing for analysis of vaccine coverage and factors affecting coverage.

The review also included all modelling studies that used pre-COVID-19 data to estimate the impacts of the COVID-19 pandemic on immunisation programmes. In addition, Qualitative studies that explored the effect of the COVID-19 pandemic on the experiences of caregivers or parents accessing childhood vaccination services and healthcare workers providing childhood vaccination services were included.

An article was deemed to have studied the correct population (Childhood immunisation programmes in West Africa) and included in the review if it showed results on at least one of the BCG, Measles, Polio, Diphtheriapertussis, Pentavalent, Meningococcal or Yellow Fever vaccines in at least one West African country. For this review, only the results from the West African country were included for analysis. There were no language restrictions, and studies not in English were run through the Google translator AI (Google Translate) and then analysed.

### Exclusion criteria

This study excluded systematic reviews, modelling studies that excluded pre-COVID-19 vaccination data, and papers that did not estimate the impact of the COVID-19 pandemic on childhood vaccine coverage.

### Information sources and search strategy

OCO and VAO conducted a literature search in the PubMed, Embase, Scopus, and Web of Science databases and filtered it to allow only papers published between January 2020 and May 2022. The search strategy explored alternate search words for the main question items and used British and American spellings where necessary. Truncation and quotes were used to broaden the search parameters. Boolean commands “AND” and “OR” were used to combine search groups ().

### Study records/screening

Search results from the respective databases were saved, downloaded, and imported into the Rayyan (www.rayyan.ai) web-based data management software.^36^

Articles were deduplicated in Rayyan and manually reviewed by OCO and VAO. Titles and abstracts were screened based on the inclusion criteria. After that, a full-text screening of the remaining articles on Rayyan was performed. Agreement on article inclusion between OCO and VAO was required before being included for further analysis. The included articles had their references screened to determine if any relevant citations were suitable for inclusion.

### Data management

**Figure 2:**
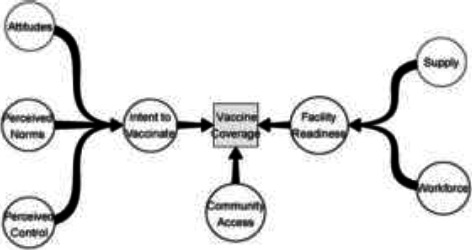
Conceptual framework for vaccine coverage.^37^

Following full-text screening and review, relevant data were extracted using a form created in Excel by one reviewer- OCO. For quantitative data, the data extracted included author, title, year of publication, study design, study population, length of study, inclusion criteria met by the paper, study aim, vaccine and vaccine characteristics studied, and data collection mode. The form also extracted the included studies' results, statistical methods, conclusions, and recommendations. For Qualitative data, the data extracted included author, title, year of publication, study population, length of study, study aim, vaccine and vaccine characteristics studied, data collection mode (KIs, FGD), major themes that emerged from the paper, conclusions, and recommendations by the article. Data analysis was based on the framework of Phillips et al. on factors determining vaccine coverage and contributing to inequalities in vaccine access (Figure 2).^37^ For quantitative studies, proportions and differences in vaccination numbers were extracted from the studies and analysed by country. In contrast, qualitative studies were analysed along thematic lines identified by the authors. All forms of studies were analysed following the conceptual framework for vaccination coverage (Figure 2). 37

### Quality assessment

Quality for quantitative studies was assessed at the study design level to determine the presence or absence of bias during data collection, reporting, and management, including missing data handling, appropriate use of relevant statistical tests and methods, and data interpretation. Quality assessment for the qualitative studies was carried out using the four criteria described by Lincoln et al.^38^,^39^ These criteria include credibility, dependability, confirmability, and transferability.

These quality assessment criteria were used because they allowed for a more flexible quality assessment in the dynamic context of the COVID-19 Pandemic.

## Results

**Figure 3:**
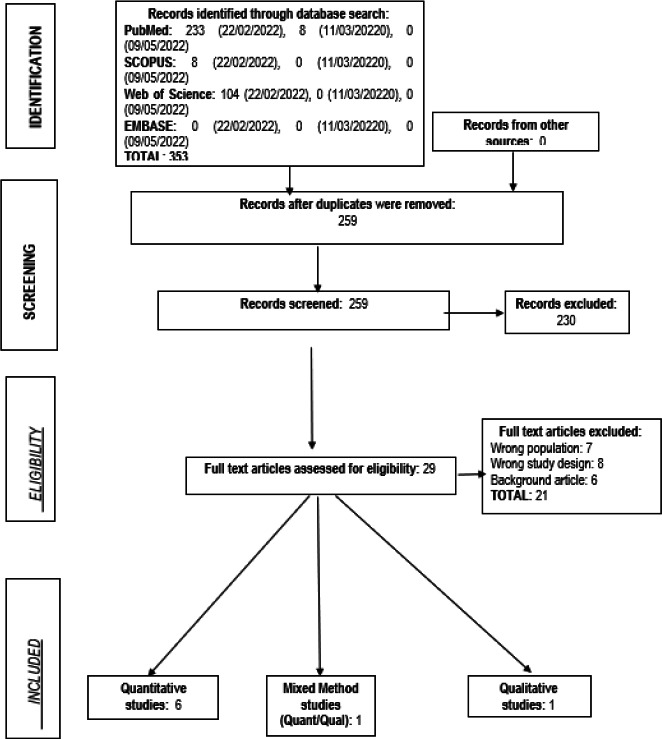
PRISMA flowchart of the systematic search process

Three hundred fifty-three (353) publications from four databases were retrieved. Retrieved results included 233, 8, 104 and 0 from PubMed, Scopus, Web of Science and Embase, respectively, on 22/02/2022. Eight results were retrieved from PubMed on 11/03/2022, while the other databases returned no additional results. The databases returned no other results at the last search session on 09/05/2022. Following deduplication, 259 studies were screened, and 230 publications were excluded due to a wrong publication type/irrelevant study. The full texts of 29 studies were retrieved and screened for inclusion. On full-text screening, twenty-one articles were excluded based on a wrong study population, study design or being a background article, e.g., editorials or letters to the editor. Eight studies were finally included in this review, and a search of the references in the selected articles did not reveal any paper relevant to this review (Figure 3).

### Study characteristics

The review included six quantitative studies on vaccination coverage trends before and during the COVID-19 pandemic 40–45, one qualitative study on the utilisation of immunisation services during the COVID-19 pandemic^46^, and one mixed method-quantitative and qualitative study- on vaccination coverage and the utilisation of immunisation services.^47^ Data from eight of 17 West African countries were included in this review: Ghana.^42^,^47^, Guinea^42^, Liberia^44^,^45^, Mali^45^, Niger^40^, Nigeria^42^,^45^,^46^, Senegal^42^,^43^, Sierra Leona.^41^,^45^

### Quantitative Studies

The tables below summarise quantitative data extracted along individual vaccine lines from the eligible studies.

**Table 1:** Summary of quantitative studies

1^st^ Author/Year	Type Of Study	Country Studied	Vaccine Studied	Period Of Study	Sample Size	Data Source	Type Of Analysis	Results
Sow A. 2020	Retrospective quantitative analysis	Senegal	Bcg, oral polio 1,2,3, pentavalent 1,2,3, measles, yellow fever	Mar 2018-Aug 2020	>5000	Local hospital vaccination records	Simple proportions	23% reduction in vaccines given at birth
Bimpong K. 2021	Mixed methods study-retrospective quantitative data analysis, thematic qualitative analysis	Ghana	Bcg, pentavalent 1,2, measles	Apr 2019-May 2020	8000	Local hospital vaccination records, HCWS and caregivers	Simple proportions	BCG: -(47%) Penta1: -(42%) Penta2: -(40.3%) Penta3: -(39%) Mcv1: -(10.5%) Mcv2: -(0,9%)
Mairama Baissa 2020	Retrospective quantitative data analysis of aggregated health data on utilisation of healthcare services	Niger	Penta 1, 3, var1	Jan 2019 -Jun 2020	Na	NHIS, OPD registers, MOH data sources	Simple proportions. Wilcoxon signed-rank tests.	Penta1: -49%(-58, -40) Penta3: -48% (-57, -39)
Buonsenso 2020	Retrospective data analysis of vaccination rates using hospital records	Sierra leone	Bcg, oral polio 1,2,3, pentavalent 1,2,3, measles, yellow fever	Mar 2019-apr 2020	Na	Local hospital records	Wilcoxon ranked tests	**ERROR! REFERENCE SOURCE NOT FOUND.**
Balcha Maresha 2021	Retrospective data analysis of immunisation volumes	Guinea, Ghana, Nigeria, Senegal	Dpt, mcv1 and 2	Jan 2019-Jun 2020	Na	Administrative data records	Simple proportions	
								COUNTRY
								Ghana
								Guinea
								Liberia
								Mali
								Niger
								Nigeria
								Senegal
								Sierra Leone
								**Table 3**
Emilia Connoly 2022	Retrospective mixed methods study (quantitative and modelling)	Liberia	Bcg, oral polio 0,1,2,3, pentavalent 1,2,3 measles	Jan 2016-Aug 2021	Na	Health information records	Negative binomial regression	
								VACCINE
								**BCG**
								**OPV0**
								**OPV1**
								**PENTA1**
								**OPV2**
								**PENTA2**
								**OPV3**
								**PENTA 3**
								**MCV1**
								**YELLOW FEVER**
								**MCV2**
								**Table 5**
Shapira Gil 2021	C19 disruptions to matches in sub-Saharan Africa	Liberia, Mali, Nigeria, Sierra Leone	Bcg and pentavalent 3	Jan 2018 – Feb 2020	Na	Health administrative data	Interrupted time series, Ordinary least squares	Table 4

*95% Ci provided where available

**Table 1:** Summary table of reductions in immunisation volumes in percentages from March 2020 – August 2021 showing national and subnational figures

COUNTRY	BCG	OPV0	OPV1	PENT1/DPT1	OPV2	PENT2/DPT2	OPV3	PENT3/DPT3	MEASLES	YELLOW FEVER
Ghana#	-47			-42		-40				
Ghana								-4	-4	
Guinea								-52	-53	
Liberia	0.3 (-5.2, -5.8)	-11	-33	-16	-38	-24	-39	-7.8 (-13.1, -2.5)	-1	
Mali	-11.8 (-15.4, -8.2)							-17.4(-22.6,12.3)		
Niger				-49 (-8, -40)		-48 (-57, -39)		-48		
Nigeria								-12	-13	
Senegal								-14	-5	
Sierra leone#	-53	-53	-71	-71	-79	-79	-78	-78	-66	-66
Sierra leone	-7.4							-12.6 (-19.1, -6.1)	-7.4 (-11.9, -2.9)	

# Data From Subnational Data, E.G., Regional/State Hospital Records

*Blank Cells- No Reported Data From The Literature Reviewed

*Bcg-Bacille Calmette Guerin, Opv- Oral Polio Vaccine, Pent- Pentavalent, Dpt-Diphtheria Pertussis Tetanus

*95% Cis Provided Where Available

**Table 2:** 2019 National infant childhood immunisation coverage estimates in percentages before the pandemic. WHO-UNICEF estimates.^48^

COUNTRY	BCG	DPT1	DPT3	MCV1	OPV3	YELLOW FEVER
Ghana	**96**	**97**	**97**	**92**	**97**	**92**
Guinea	**85**	**85**	**47**	**47**	**82**	**80**
Liberia	**84**	**94**	**87**	**85**	**87**	**79**
Mali	**83**	**82**	**71**	**70**	**54**	**66**
Niger	**70**	**92**	**81**	**79**	**81**	**83**
Nigeria	**67**	**65**	**57**	**54**	**57**	**-**
Senegal	**103**	**106**	**100**	**96**	**100**	**89**
Sierra Leone	**75**	**95**	**95**	**87**	**95**	**90**

**Table 3:** Routine DPT3 And MCV immunisation performance In West African countries during the pandemic.^42^

COUNTRY	MONTHLY MEAN DPT3 VACCINATION			MONTHLY MEAN MCV1 VACCINATION		
	JAN-MAR 2020	APRIL-JUN 2020	% CHANGE IN THE TWO QUARTERS	JAN-MAR 2020	APRIL-JUN 2020	% CHANGE IN THE TWO QUARTERS
Ghana	95358	919678	-4	93035	89443	-4
Guinea	35352	16850	-52	35605	16788	-53
Nigeria	560428	492483	-12	523869	455412	-13
Senegal	51663	44539	-14	41719	39486	-5

**Table 4:** Disruptions In BCG and PENTA3 vaccine coverage in West African countries during COVID-19.^45^

COUNTRY	TOTAL % SHORTFALL IN BCG VACCINATIONS APR-JUN 2020	TOTAL % SHORTFALL IN PENTA 3 VACCINATIONS APR-JUN 2020
Liberia	0.3	-7.8
Mali	-11.8	-17.4
Nigeria	-4.1	-9.0
Sierra Leone	-7.4	-12.6

**Table 5:** Childhood immunization during The COVID-19 pandemic In Liberia. (Adapted from Connoly et al. 44)

VACCINE	MAR-AUG 2020 (A)			SEPT 2020-FEB 2021 (B)			% TOTAL CHANGE
	EXPECTED	OBSERVED	% DIFF (95% PI)	EXPECTED	OBSERVED	% DIFF (95% PI)	
BCG	1220	1123	-8 (-24.5-10.7)	884	1129	+28.1 (6.7-56.5)	+0.5
OPV0	1016	927	-8.8 (-23-7.4)	729	832	+14.1 (-4.3-35.6)	-11.4
OPV1	1369	1198	-12.5 (-25.1-4)	1144	1774	+55.1 (32.8-83.3)	+32.5
OPV2	1274	1062	-16.6 (-34.9-0.3)	1076	1707	+56.5 (26.3-96.3)	+37.8
OPV3	1333	1041	-21.9 (-35.4-7.9)	1199	1717	+43.1 (18.9-72.2)	+39.4
PENTA1	1360	1190	-12.5 (-25.5-1)	1149	1423	+23.8 (6.3-46.3)	+16.4
PENTA2	1287	1063	-17.4 (-31.5- -2.5)	1097	1398	+27.4 (6.8-53.6)	+24
PENTA3	1348	1035	-23.2 (-36.5-9.2)	1216	1492	+22.7 (0.5-44.4)	+30.7


**Table 6:** Child healthcare and immunisations in Sierra Leone during the pandemic (Adapted from Buonsenso et al. 41)

VACCINE	MAR-APR 2019	MAR-APR 2020	%CHANGE
**BCG**	36	17	-52.7
**OPV0**	36	17	-52.7
**OPV1**	58	17	-70.7
**PENTA1**	58	17	-70.7
**OPV2**	71	15	-78.9
**PENTA2**	71	15	-78.9
**OPV3**	67	15	-77.6
**PENTA 3**	67	15	-77.6
**MCV1**	64	22	-65.6
**YELLOW FEVER**	64	22	-65.6
**MCV2**	49	8	-83.7

**Table 7:** Summary of qualitative studies

1^ST^ AUTHOR/YEAR	TYPE OF STUDY	COUNTRY	COMMON THEMES	PERIOD OF INTEREST	POPULATION/SAMPLE SIZE	DATA SOURCE
**Ahmed T. 2021**	Contextual narrative synthesis of the utilisation of basic and advanced MNCHC services	NIGERIA	Fear of infection and COVID-19 disruption of routine activities and livelihoods A lack of safety protection and logistical support for healthcare workers	April/May 2019 and April/May 2020	NA	Hospital Records of a Nigerian Tertiary Hospital
**Bimpong K. 2021**	Mixed methods study-Retrospective Quantitative data analysis, Thematic qualitative analysis	GHANA	HEALTH CARE WORKERS: Fear Misinformation CLIENTS Fear Side effects	Apr 19-May 20	8000	Hospital vaccination records, HCWs and Caregivers

**Table 8:** Quality analysis

AUTHOR	DATA COLLECTION	Was the sample representative	DATA REPORTING	DATA MANAGEMENT	TEMPORAL TRENDS	STATISTICAL METHODS USED	RESULT REPORTING
**Sow A. 2020**	Probable reporting bias	State representative sample	Inadequate. It does not provide individual data for individual vaccines	No mention of handling missing data	Moderately adequate. Compared to 2 years of data	No tests of significance	Appropriate
**Bimpong K. 2021**	Probable reporting bias	State representative sample	Adequate	Mentioned missing data but did not say how it was accounted for in the analysis	Poor. Compared to one year	Wilcoxon signed-rank test	Appropriate
**Mairama Baissa 2020**	Probable reporting bias	State representative sample	Adequate	No mention of handling missing data	Poor. Compared to one year of data	T-test, Chi square, Anova, Bonferroni	Appropriate
**Ahmed T. 2021**	Probable reporting bias	State representative sample	NA	NA	NA	NA	NA
**Business 2020**	Probable reporting bias	Local county representative sample	Adequate	No mention of handling missing data	Poor. Compared to one year of data.	Wilcoxon signed-rank test	Appropriate
**Balcha Maresha 2021**	Probable reporting bias	Nationally representative sample	Adequate	No mention of handling missing data	Moderately adequate. Compared to 2 years of data	No tests of significance	Appropriate
**Emilia Connolly 2022**	Probable reporting bias	Nationally representative sample	Adequate	Managed missing data appropriately. Missing data was treated as “missing at random.”	Highly adequate. Compared to 6 years of data	Binomial regression	Appropriate
**Shapira Gil 2021**	Probable reporting bias	Nationally representative sample	Adequate	Managed missing data appropriately. Missing data was treated as “missing at random.”	Moderately adequate. Compared to 2 years of data	ITS, OLS	Appropriate

### Qualitative studies

Two qualitative studies were included in this study.^62, 63^ and were analysed along thematic lines based on Phillips et al.'s framework for the determinants of vaccination coverage 55

### Quality analysis

At the study design level, all quantitative studies relied on data collected via registries of data reports from various national registries. Only two studies^44^,^45^ Explicitly stated how missing data were managed. One study^47^ Mentioned missing data but did not state how this was handled. Other studies were silent about missing data.

Sample sizes were not explicitly stated for all studies. However, one study^41^ had a sample representative of a county or locality, three studies^40^,^43^,^46^ had state or regionally representative samples, and four studies^40^,^42^,^44^,^45^ Had nationally representative samples. Seven studies provided adequate data reports, while one study^43^ did not provide individual data for the reported vaccine.

Both qualitative studies showed some quality as the papers used appropriate research processes/procedures to answer the questions. However, while Bimpong^47^ detailed the sampling procedure and process, Ahmed^46^ did not provide any information about the sampling process or procedure, so the representability of his sample could not be determined. Furthermore, neither study was explicit on the methodology or framework used for their chosen approaches, nor were their methods fully justified. Bimpong provided some information about the qualitative process to replicate their study if necessary and utilised quotes to aid descriptions and findings compared to Ahmed.^46^

## Discussion

**Figure 4:**
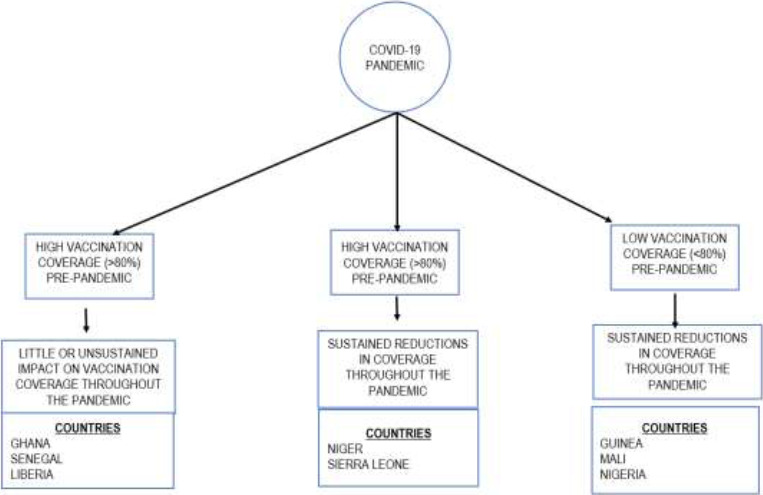
Conceptualising the impact of the COVID-19 pandemic on childhood immunisation programmes in studied West African Countries

The impact of the COVID-19 pandemic on West African countries' immunisation programmes can be described along three major themes (Figure 4). The most significant changes in vaccination volumes (>50%) occurred in April 2020 across the eight countries in this review. However, these changes in April may not have been solely due to the direct effects of the COVID-19 pandemic because this period coincided with the WHO directive recommending the suspension of mass immunisation programmes and the reduction of routine immunisation programmes due to concerns about the transmission of the Covid-19 virus.^30^ However, the continued changes to immunisation coverage noted in this review may be attributed directly to the pandemic alongside other factors like the strength of the health system before COVID-19 and the successes of the childhood immunisation programme before the pandemic. All of these contributed to the varying impact of the COVID-19 pandemic on childhood immunisation programmes in West African countries by impacting critical components of the vaccination cycle and determinants of vaccination coverage.^37^,^49^

Countries with high pre-pandemic vaccination coverage, like Ghana, Senegal and Liberia, experienced minimal (<20%) and short-lived changes to vaccination volumes during the COVID-19 pandemic. However, measles outbreaks were still common in these countries, pointing to local contextual factors that may have pre-existed and still exist despite the high coverage reported.^42^,^50^–^53^ Countries like Niger and Sierra Leone reported high vaccine coverage before the COVID-19 pandemic but witnessed reductions in immunisation during the COVID-19 pandemic lasting longer than August 2020.

However, these pre-pandemic gains may have been lost during the COVID-19 pandemic due to pre-existing high levels of insecurity, political unrest, poor vaccine storage and distribution, and a lack of a sustainable health system resilience plan combined with the effects of the COVID-19 pandemic. Other countries in this review- Nigeria, Mali, and Guinea- have poor pre-pandemic vaccine coverage and have demonstrated reduced vaccination coverage throughout the COVID-19 pandemic. The reported reductions in coverage were possibly due to the amplification of already existing inequalities in vaccine distribution and utilisation, pre-existing security challenges and political instability.

Five of the eight countries (Guinea, Mali, Nigeria, Niger, Sieera-Leone) in this review had significant reductions (>50%) in childhood vaccination coverage during the COVID-19 pandemic. When these reductions are viewed in light of the pre-existing challenges in these countries, the potential national and regional impacts on childhood health become enormous. While these disruptions' medium to long-term effects may not be known, the shortterm effects are currently being reported. The WHO says that a resurgence in vaccine-preventable diseases is rising in Africa.^54^ In 2022, between January and March, 17,500 cases (400% increase) of measles were reported from 20 African countries, eight more countries compared to the same period in 2021.^54^

The West African region contributed about 44% of measles cases (Nigeria 5,613, Cote d'Ivoire 1,075, Mali 946 contributing the most cases).^55^ Twenty African countries reported a variant of polio in 2020, which increased to Twenty-four in 2021.^54^ While all African countries except Malawi have been certified wild polio virus-free, the continent still has vaccine-derived poliovirus to contend with^54^ Ten countries in West Africa have circulating vaccine-derived poliovirus infections. Only Togo and Ghana are polio-free in the region. The reduction of vaccinations caused by the COVID-19 pandemic is capable of causing the cross-border spread of vaccine-derived poliovirus, leading to a regional poliovirus crisis and worsening the poliovirus epidemics within West African Countries.

Hence, it is imperative that in the short term, catch-up immunisation programmes to provide the missed doses of vaccines be done in countries that experienced severe disruptions. These countries may also employ more National Immunisation days and Supplemental Immunisation activities to improve vaccine coverage. In the medium to long term, strengthening the facility readiness, improving community access, and increasing the intent to vaccinate while strengthening health system resilience should be pursued. Facility readiness, described as the ability of the existing health system to supply vaccines to meet demand, can be influenced by varying factors.

An Important factor influencing facility readiness is the government's political will and the provision and management of funding.^56^

This review showed that programmes with a history of strong political and governmental support and good funding, e.g., Senegal, perform better than programmes with poor funding, poor political will, and government support, e.g., Guinea. Another factor directly affecting facility readiness and funding is the provision of adequate vaccine distribution, supply and storage.^57^ Distribution, supply, and storage are essential to prevent stock-outs and ensure that a facility continues offering vaccine services. Proper distribution and storage also ensure that the vaccine provided to children is high-quality. The availability of trained healthcare workers in adequate numbers is also pivotal to a facility's success in providing vaccine services.

The ability to bridge the facility's readiness to provide immunisation services and the intent to vaccinate depends on the community's access to these services. The prevailing social determinants of health, such as socioeconomic status, location, race, sex, and age distribution, the absence or presence of advocacy for the use of immunisation services, the absence of poor community partnerships, the attitude of the community towards health-seeking, and the perceived quality of care in the health facility, determine this access.^39^,^58^–^60^ Contextualised bottom-up approaches like the community-driven comprehensive national immunisation programme in Senegal or the community engagement, social mobilisation and communication strategy of Liberia provide room for end-users ownership of the health system and improve community access to vaccines.^49^,^61^

### Implications for policy

Health shocks like the COVID-19 pandemic are on the rise. Therefore, policies that strengthen the available health system are imperative to prevent these shocks' effects on the health system. Policies that improve facility readiness- improving community access to vaccines, storage and distribution of vaccines—and better government funding and support are essential to withstand the effects of health shocks.

### Limitations of the review

A limitation of this review is that the currently available evidence is few and heterogeneous. Except for DPT3 and MCV vaccines, not all vaccines were studied for all countries, making estimating the pandemic's effect on childhood immunisation programmes within and between the countries along respective vaccine lines challenging.

## Conclusion

The pandemic may have amplified pre-existing inequalities in vaccine access and system weakness, which hampered the provision of vaccination services during the pandemic. While the changes in the immunisation programmes were significant and lasted longer in some countries like Nigeria, Guinea, Mali, Sierra-leone and Niger, they were less substantial and short-lived in others like Ghana, Senegal and Liberia, emphasising the fact that the effect of the pandemic was unique to each country. Pandemics like the COVID-19 pandemic can damage the existing fragile health systems of LMICs and erode the gains of universal health coverage and immunisation. Improving health system resilience is critical to ensuring that the gains in vaccination and universal healthcare are not eroded by public health shocks, as shown by Niger and Sierra Leone. Therefore, while improving facility readiness, community access and intent to vaccinate, attention must be paid to processes and policies that strengthen health systems and prepare them for future shocks.

## References

[1] World Health Organisation (2022). Essential programme on immunisation [Internet].

[2] World Health Organization (1994). Ninth general programme of work covering the period 1996-2001.

[3] Galazka AM (1993). Expanded programme on immunization: the immunological basis for immunization. Module 2: diphtheria. WHO document WHO. EPI/GEN/93.12.

[4] Kim-Farley RJ (1992). EPI for the 1990s. The Expanded Programme on Immunization Team. Vaccine.

[5] World Health Organization (1998). Global Programme for Vaccines and Immunisation, Expanded Programme on Immunisation. Immunization Policy WHO/EPI/Geneva/9503, Rev.

[6] LaForce FM, Konde K, Viviani S, Préziosi M-P (2007). The meningitis vaccine project. Vaccine.

[7] LaForce FM, Okwo-Bele J-M (2011). Eliminating epidemic group A meningococcal meningitis in Africa through a new vaccine. Health Affairs.

[8] World Health Organisation (1996). Field Guide for supplementary activities aimed at achieving polio eradication.

[9] Birmingham ME, Aylward RB, Cochi SL, Hull HF (1997). National immunization days: state of the art. J Infect Dis.

[10] Khan S, Hancioglu A (2019). Multiple indicator cluster surveys: delivering robust data on children and women across the globe. Studies in family planning.

[11] Fabic MS, Choi Y, Bird S (2012). A systematic review of Demographic and Health Surveys: data availability and utilization for research. Bulletin of the World Health Organization.

[12] Henderson RH, Sundaresan T (1982). Cluster sampling to assess immunization coverage: a review of experience with a simplified sampling method. Bulletin of the World Health Organization.

[13] World Health Organization (2008). Training for mid-level managers (MLM): Module 7: the EPI coverage survey.

[14] Cutts FT (1998). Advances and challenges for the expanded programme on immunization. British medical bulletin.

[15] World Health Organization (2015). Immunization in practice: a practical guide for health staff.

[16] World Health Organisation (2020). Implementing the Immunization Agenda 2030: A Framework for Action through coordinated planning, Monitoring, and Evaluation, Ownership and Accountability, and Communications and Advocacy.

[17] World Health Organisation (2022). Vaccines and Immunisations [Internet]. Vaccines and Immunisations.

[18] Casey RM, Hampton LM, Anya B-PM, Gacic-Dobo M, Diallo MS, Wallace AS (2017). State of equity: childhood immunization in the World Health Organization African Region. Pan Afr Med J.

[19] Galles NC, Liu PY, Updike RL, Fullman N, Nguyen J, Rolfe S (2021). Measuring routine childhood vaccination coverage in 204 countries and territories, 1980–2019: a systematic analysis for the Global Burden of Disease Study 2020, Release 1. The Lancet.

[20] Bobo FT, Asante A, Woldie M, Dawson A, Hayen A (2022). Child vaccination in sub-Saharan Africa: Increasing coverage addresses inequalities. Vaccine.

[21] Mondiale de la Santé WHO (2019). Meeting of the Strategic Advisory Group of Experts on immunization, April 2019–conclusions and recommendations. Weekly Epidemiological Record.

[22] World Health Organisation (2020). Pulse survey on continuity of essential health services during the COVID-19 pandemic-Interim report, 27th August 2020.

[23] World Health Organisation (2022). Third round of the national pulse survey on continuity of essential health services during the COVID-19 pandemic: interim report, 7th February 2022.

[24] World Health Organization (2021). Second round of the national pulse survey on continuity of essential health services during the COVID-19 pandemic: interim report, 22nd April 2021.

[25] World Health Organisation (2020). At least 80 million children under one are at risk of diseases such as diphtheria, measles and polio as COVID-19 disrupts routine vaccination efforts, warn Gavi, WHO and UNICEF.

[26] GAVI-The Vaccine Alliance (2020). COVID-19: massive impact on lower-income countries threatens more disease outbreaks [Internet].

[27] World Health Organisation (2022). WHO Immunization Data Portal [Internet].

[28] Mercado Marxie, United Nations Children Fund (2020). Geneva Palais briefing note on the impact of COVID-19 mitigation measures on vaccine supply and logistics.

[29] United Nations Children Fund (2020). Impact of COVID-19 on Vaccine Supplies.

[30] World Health Organisation (2020). Guiding Principles for Immunization activities during the COVID-19 pandemic.

[31] Adeloye D, Jacobs W, Amuta AO, Ogundipe O, Mosaku O, Gadanya MA (2017). Coverage and determinants of childhood immunization in Nigeria: A systematic review and meta-analysis. Vaccine.

[32] Isba R, Edge R, Jenner R, Broughton E, Francis N, Butler J (2020). Where have all the children gone? Decreases in paediatric emergency department attendance at the start of the COVID-19 pandemic of 2020. Archives of Disease in Childhood.

[33] Galadima AN, Zulkefli NAM, Said SM, Ahmad N (2021). Factors influencing childhood immunisation uptake in Africa: a systematic review. BMC Public Health.

[34] Chandir S, Siddiqi DA, Setayesh H, Khan AJ (2020). Impact of COVID-19 lockdown on routine immunisation in Karachi, Pakistan. The Lancet Global Health.

[35] Moher D, Liberati A, Tetzlaff J, Altman DG, Prisma Group (2009). Preferred reporting items for systematic reviews and meta-analyses: the PRISMA statement. BMJ.

[36] Ouzzani M, Hammady H, Fedorowicz Z, Elmagarmid A (2016). Rayyan—a web and mobile app for systematic reviews. Systematic reviews.

[37] Phillips DE, Dieleman JL, Lim SS, Shearer J (2017). Determinants of effective vaccine coverage in low and middle-income countries: a systematic review and interpretive synthesis. BMC Health Services Research.

[38] Lincoln YS, Guba EG (1986). But is it rigorous? Trustworthiness and authenticity in naturalistic evaluation. New Directions for Program Evaluation.

[39] Stenfors T, Kajamaa A, Bennett D (2020). How to assess the quality of qualitative research. The Clinical Teacher.

[40] Abdoulaye MB, Oumarou B, Moussa H, Anya BM, Didier T, Nsiari-Muzeyi BJ (2021). The impact of the COVID-19 pandemic on health service utilisation in the City of Niamey: a study conducted in 17 healthcare facilities from January to June 2020. Pan Afr Med J.

[41] Buonsenso D, Cinicola B, Kallon MN, Iodice F (2020). Child Healthcare and Immunizations in Sub-Saharan Africa During the COVID-19 Pandemic. Front Pediatr.

[42] Masresha B, Luce R, Katsande R, Dosseh A, Tanifum P, Lebo E (2021). The impact of the COVID-19 pandemic on measles surveillance in the World Health Organisation African Region, 2020. Pan Afr Med J.

[43] Sow A, Gueye M, Boiro D, Ba A, Ba ID, Faye PM (2020). Effect of COVID-19 on routine immunization schedule in Senegalese hospitals. The Pan African Medical Journal.

[44] Connolly E, Boley EJ, Fejfar DL, Varney PF, Aron MB, Fulcher IR (2022). Childhood immunization during the COVID-19 pandemic: experiences in Haiti, Lesotho, Liberia and Malawi. Bull World Health Organ.

[45] Shapira G, Ahmed T, Drouard SHP, Amor Fernandez P, Kandpal E, Nzelu C (2021). Disruptions in maternal and child health service utilization during COVID-19: analysis from eight sub-Saharan African countries. Health Policy Plan.

[46] Ahmed T, Rahman AE, Amole TG, Galadanci H, Matjila M, Soma-Pillay P (2021). The effect of COVID-19 on maternal newborn and child health (MNCH) services in Bangladesh, Nigeria and South Africa: call for a contextualised pandemic response in LMICs. International Journal for Equity in Health.

[47] Bimpong KA, Nuertey BD, Seidu AS, Ajinkpang S, Abdul-Mumin A (2021). Decline in Uptake of Childhood Vaccinations in a Tertiary Hospital in Northern Ghana during the COVID-19 Pandemic. BioMed research international.

[48] World Health Organisation (2020). WHO and UNICEF estimates of National Infant Immunisation coverage [Internet]. Immunization Analysis and Insights.

[49] Dixit SM, Sarr M, Gueye DM, Muther K, Yarnko TR, Bednarczyk RA (2021). Addressing disruptions in childhood routine immunisation services during the COVID-19 pandemic: perspectives from Nepal, Senegal and Liberia. BMJ Glob Health.

[50] Mbengue MAS, Sarr M, Faye A, Badiane O, Camara FBN, Mboup S (2017). Determinants of complete immunization among senegalese children aged 12–23 months: evidence from the demographic and health survey. BMC Public Health.

[51] Sarker AR, Akram R, Ali N, Chowdhury ZI, Sultana M (2019). Coverage and Determinants of Full Immunization: Vaccination Coverage among Senegalese Children. Medicina.

[52] Ameyaw EK, Kareem YO, Ahinkorah BO, Seidu A-A, Yaya S (2021). Decomposing the rural-urban gap in factors associated with childhood immunisation in sub-Saharan Africa: evidence from surveys in 23 countries. BMJ Global Health.

[53] United Nations Children Fund (2021). Country Office Annual Report 2021 [Internet].

[54] World Health Organisation (2022). Vaccine-preventable disease outbreaks are on the rise in Africa. [Internet].

[55] Centre for Disease Control and Prevention (2022). Global Measles outbreaks [Internet]. Global Immunization.

[56] Naimoli JF, Challa S, Schneidman M, Kostermans K (2008). Toward a grounded theory of why some immunization programmes in sub-Saharan Africa are more successful than others: a descriptive and exploratory assessment in six countries. Health policy and planning.

[57] Haddad S, Bicaba A, Feletto M, Taminy E, Kabore M, Ouédraogo B (2009). System-level determinants of immunization coverage disparities among health districts in Burkina Faso: a multiple case study. BMC international health and human rights.

[58] Rainey JJ, Watkins M, Ryman TK, Sandhu P, Bo A, Banerjee K (2011). Reasons related to non-vaccination and under-vaccination of children in low and middle-income countries: findings from a systematic review of the published literature, 1999–2009. Vaccine.

[59] Jani JV, De Schacht C, Jani IV, Bjune G (2008). Risk factors for incomplete vaccination and missed opportunity for immunization in rural Mozambique. BMC Public Health.

[60] Fatiregun AA, Okoro AO (2012). Maternal determinants of complete child immunization among children aged 12–23 months in a southern district of Nigeria. Vaccine.

[61] Enria L, Bangura JS, Kanu HM, Kalokoh JA, Timbo AD, Kamara M (2021). Bringing the social into vaccination research: Community-led ethnography and trust-building in immunization programs in Sierra Leone. PLoS One.

